# Spontaneous Epidural Hematoma in Sickle Cell Crisis: A Case Report

**DOI:** 10.7759/cureus.24492

**Published:** 2022-04-26

**Authors:** Jiss Joy, Maria A Vasnaik, Vivek Bhat, Seetharam Anandram, Arun George

**Affiliations:** 1 Internal Medicine & Hematology, St. John’s Medical College, Bangalore, IND; 2 Internal Medicine & Hematology, St. John's Medical College, Bangalore, IND; 3 Radiology, St. John's Medical College, Bangalore, IND

**Keywords:** stroke, neurologic complication, sickle cell crisis, hemoglobinopathy, intracranial bleed

## Abstract

Epidural hematoma (EDH) classically occurs secondary to trauma. Spontaneous EDH is uncommon and can be a rare complication of sickle cell disease (SCD). We report the case of a 20-year-old Indian male with sickle cell anemia, who presented with a sickling bony crisis and suffered a non-traumatic EDH within 24 hours of admission.

A 20-year-old male presented with generalized body pain, suggestive of a sickling bony crisis. He was promptly admitted and received standard treatment for the same. The next day, he developed severe right-sided headache, associated with orbital pain, decreased movements on the right side, and altered sensorium. He had a Glasgow coma scale score of 8/15, and reduced power of the right upper limb and lower limb. Computed tomography (CT) and magnetic resonance imaging (MRI) of the brain showed a left-sided large parieto-temporal epidural hematoma with midline shift and mass effect. He underwent emergency decompressive craniotomy and evacuation of the hematoma, following which he recovered well, with no residual deficits.

Spontaneous EDH is being increasingly reported in SCD. Possible mechanisms include skull bone infarction, altered skull bone anatomy due to extramedullary hematopoiesis, and venous congestion due to sluggish blood flow in diploic veins. In our patient, altered skull anatomy appeared to be the causative mechanism. Early identification of EDH and aggressive neurosurgical management is crucial to survival and a good prognosis.

## Introduction

Acute epidural hematoma (EDH) is classically associated with trauma. Non-traumatic or spontaneous EDH is an uncommon occurrence, with implicated etiologies including infections, coagulopathies, advanced renal disease, and malignancy, among others [1]. While cerebrovascular events, particularly ischemic stroke, are common in sickle cell disease (SCD) [2,3], spontaneous EDH secondary to SCD is rare, with only a few reports in English literature [4].

We report spontaneous EDH in a young adult Indian male with SCD, who initially presented with a sickling bony crisis.

## Case presentation

A 20-year-old Indian male, born to a non-consanguineous family, who was a known case of sickle cell anemia with multiple past admissions for sickling crises, presented to the emergency department with complaints of generalized body pains. He had no other systemic complaints. On examination, he was vitally stable and had severe pallor. Under suspicion of sickling bone crises, he was admitted, and given appropriate analgesia, hydroxyurea, and intravenous fluids.

On admission, his basic hematologic and biochemical parameters were largely within normal limits, as seen in Table 1.

**Table 1:** Basic hematologic and biochemical laboratory investigations at admission TLC - total leukocyte count; INR - international normalized ratio; aPTT - activated partial thromboplastin time; AST - aspartate transaminase; ALT - alanine transaminase; ALP - alkaline phosphatase; GGT - gamma glutamyl transferase

Investigation	Patient value	Reference value
Hemoglobin	9.8 g/dL	12-16 g/dL
TLC	23,040/mm^3^	4,000-11,000/mm^3^
Platelets	213,000/mm^3^	150,000-400,000/mm^3^
INR	1.33	0.8-1.1
aPTT	30.10 seconds	26-35 seconds
Urea	90.1 mg/dL	19.0-44.0 mg/dL
Creatinine	0.56 mg/dL	0.72-1.25 mg/dL
Total bilirubin	4.39 mg/dL	0.2-1.2 mg/dL
Direct bilirubin	3.76 mg/dL	0.2-0.7 mg/dL
AST	81 U/L	5-34 U/L
ALT	19 U/L	5-34 U/L
ALP	402 U/L	48-95 U/L
GGT	12 U/L	9-36 U/L
Sodium	135 mEq/L	136-145 mEq/L
Potassium	4.5 mEq/L	3.5-5.1 mEq/L
Chloride	101 mEq/L	98-107 mEq/L

**Figure 1:**
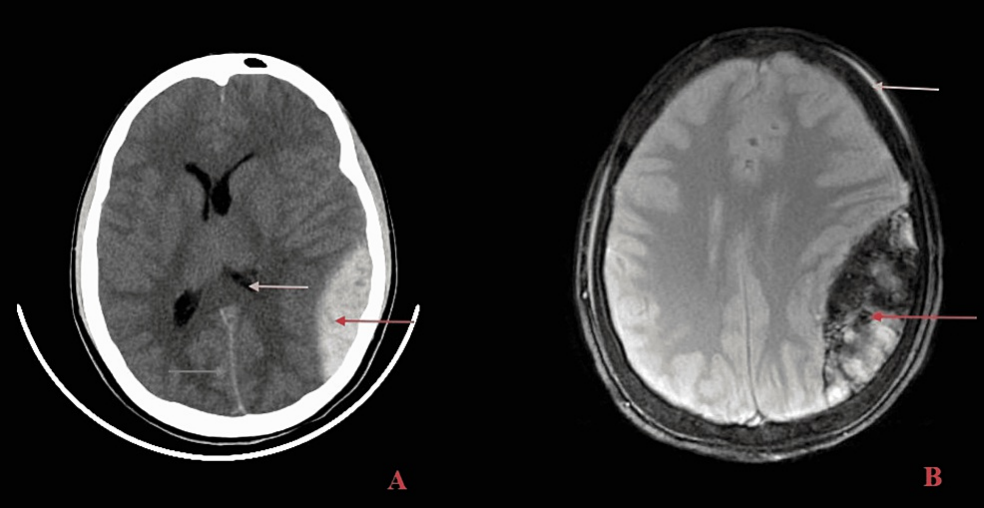
A. CT brain axial section showing a biconvex hematoma, suggestive of acute EDH (red arrow), with midline shift to the right (blue arrow), and effacement of the left lateral ventricle (yellow arrow). B. MRI brain axial section showing acute and ongoing EDH (red arrow). Diffuse cerebral edema is present, and mild scalp contusion is noted over the left parietal region (yellow arrow). CT - computed tomography; EDH - epidural hematoma; MRI - magnetic resonance imaging

The very next day, he developed a sudden-onset right-sided headache. This was associated with orbital swelling, neck stiffness, decreased movements on the right side of the body, and several bouts of projectile vomiting. He had altered sensorium but was afebrile. On examination, he had a Glasgow coma score of 8/15. Power in the right upper and lower limbs was 3/5 on the Medical Research Council (MRC) muscle power scale, and he had a right upgoing plantar reflex. His most recent blood investigations prior to this revealed a drop in hemoglobin (9.8 g/dL to 6.0 g/dL) and platelet count (213,000/mm^3^ to 83,000/mm^3^), from his baseline. Computed tomography (CT) and magnetic resonance imaging (MRI) of the brain revealed a large biconvex hematoma in the left parietal region, with a swirl sign, suggestive of an acute and ongoing epidural hematoma. There was a midline shift of 10 mm to the right, and subfalcine herniation to the right, with effacement of the left lateral ventricle. A small contusion in the left high parietal region was also noted (Figures 1A, 1B).

An emergency left temporoparietal craniotomy was performed, and the hematoma was evacuated. His hemoglobin and platelet values further dropped to 3.7 g/dL and 26,000/mm^3 ^respectively, for which he received multiple red blood cell and platelet transfusions. He received mechanical ventilation for two days, following which he was extubated. His neurological deficits improved, and he regained full neurological function of the right side of the body with improvement in the sensorium. Ten days after surgery, he was discharged on appropriate antiepileptics, having recovered completely. He has been following up on an outpatient basis and is doing reasonably well.

## Discussion

SCD is a term referring to a group of inherited hemoglobin disorders, including sickle cell anemia [5]. India accounts for the second-highest SCD burden in the world [6]. SCD has a heterogeneous distribution across multiple regions of the country, but disproportionately affects the rural tribal populations [6,7]. Among these subgroups, the prevalence is as high as 40%, with clinical course and severity varying between them [7].

In SCD, mutations in genes encoding hemoglobin subunits result in the formation of abnormal hemoglobin molecules, referred to as ‘HbS’. When deoxygenated, HbS molecules bind together in long rigid aggregates, causing the deformation of red blood cells to a sickle-like shape [5]. Eventually, this deformation becomes permanent, with the altered membrane properties causing vaso-occlusion and hemolysis, which are central to complaints in SCD.

Neurologic manifestations are common in SCD. SCD conveys a greater risk of cerebrovascular disease, with an estimated prevalence of stroke of 4.2% [2]. Most cerebrovascular events in SCD are ischemic in nature [3]. Risk factors for intracranial bleeds in SCD include therapy-related hypertension, recent transfusion, splenic sequestration, greater hemoglobin levels, and intracranial aneurysms [3]. Subarachnoid hemorrhage is the most common type of intracranial hemorrhage, with EDH rare. However, spontaneous EDH in SCD is being increasingly recognized [3,4,8,9], albeit without clear data on its frequency or risk factors.

The pathophysiology of spontaneous EDH is not entirely understood. The three main proposed mechanisms for this entity are related to bony infarction, hematopoietic marrow expansion, and altered blood flow due to increased viscosity [4,8,9]. The first hypothesis states that infarction of the skull leads to periosteal elevation and disruption of the cortical bone margin, resulting in leakage bleeding into the epidural space. This is suggested by the coexistence of subgaleal or orbital hematomas in those with EDH due to SCD [4]. MRI of the skull commonly shows bone/ marrow hyperintensity due to edema, with contrast enhancement [9]. In the second hypothesis, expansion of hematopoietic marrow in the skull, particularly after acute drops in hemoglobin, results in similar cortical thinning and blood extravasation. CT of the skull demonstrates widened diploic spaces, and cortical thinning [9]. The last mechanism states that sluggish venous blood flow due to hyperviscosity results in venous congestion and finally, rupture [9].

Our patient had a clinical course consistent with prior reports. Spontaneous EDH in SCD is reportedly more common in male patients, particularly those of the adolescent age group. Headache and vaso-occlusive crises were the most common presenting complaint. In the vast majority of those whose primary complaint was headache, EDH was present at admission, while in those with pain crises, EDH was detected around 24 hours after admission. EDH was usually unilateral, and almost half had associated, overlying bony infarctions [4]. With rapid identification and neurosurgical referral, the prognosis is good. Coexistent coagulopathies or platelet disorder portend a grave prognosis [4].

In our patient, before acute SDH, there was a drop in hemoglobin and hematocrit levels. MRI and CT of the brain showed no evidence of bony infarction, but they did show anatomical features suggestive of chronic extramedullary hematopoiesis. While neurologic imaging showed a contusion in the left high parietal region, this was likely due to the patient hitting his head on the railing of the bed after developing altered sensorium. The lack of underlying skull fracture and the minor degree of injury sustained was unlikely to contribute to EDH. So, in our patient, the acute drop in hemoglobin causing marrow expansion may have precipitated EDH.

## Conclusions

Spontaneous EDH is a rare complication of SCD. It should be suspected in SCD patients presenting with headache, with or without neurologic deficits. This is particularly when there has been a prior, sudden drop in hemoglobin. Rapid identification and neurosurgical intervention are key to good outcomes. Further research is warranted to identify the frequency of, and predisposing factors to EDH in patients with SCD.
